# Clinical Efficacy of Temozolomide and Its Predictors in Aggressive Pituitary Tumors and Pituitary Carcinomas: A Systematic Review and Meta-Analysis

**DOI:** 10.3389/fneur.2021.700007

**Published:** 2021-06-18

**Authors:** Mei Luo, Yiheng Tan, Wenli Chen, Bin Hu, Zongming Wang, Diming Zhu, Haosen Jiao, Chengbin Duan, Yonghong Zhu, Haijun Wang

**Affiliations:** ^1^Department of Neurosurgery and Pituitary Tumor Center, The First Affiliated Hospital, Sun Yat-Sen University, Guangzhou, China; ^2^Department of Histology and Embryology, Zhongshan School of Medicine, Sun Yat-Sen University, Guangzhou, China

**Keywords:** pituitary adenoma, temozolomide, aggressive pituitary tumors, pituitary carcinomas, meta-analysis

## Abstract

**Background:** A growing number of evidences suggest that TMZ applications can generate impressive benefits for APT and PC patients. However, the definite role of TMZ for individuals remains unclarified due to the variation between studies. And the predictive factors to alter its efficacy remain debatable.

**Objective:** To evaluate the long-term effectiveness and safety profile of TMZ in the treatment of pituitary malignancies, and delineate the predictors during its clinical employment.

**Results:** A literature retrieval was conducted from online databases for studies published up to December 31, 2020. Twenty one studies involving 429 patients were identified. TMZ exhibited 41% radiological overall response rate (rORR). The biochemical response rate was determinate in 53% of the functioning subset. Two-year and 4-year survival rate were 79 and 61%, respectively. TMZ prolonged the median PFS and OS as 20.18 and 40.24 months. TMZ-related adverse events occurred in 19% of patients. Regarding predictors of TMZ response, rORR was dramatically improved in patients with low/intermediate MGMT expression than those with high-MGMT (>50%) (*p* < 0.001). The benefit of TMZ varied according to functioning subtype of patients, with greater antitumor activities in functioning subgroups and fewer activities in non-functioning sets (*p* < 0.001). Notably, the concomitant therapy of radiotherapy and TMZ significantly increased the rORR (*p* = 0.007).

**Conclusion:** TMZ elicits clinical benefits with moderate adverse events in APT and PC patients. MGMT expression and clinical subtype of secreting function might be vital predictors of TMZ efficacy. In the future, the combination of radiotherapy with TMZ may further improve the clinical outcomes than TMZ monotherapy.

## Introduction

Pituitary adenomas (PAs) are common intracranial tumors with a prevalence of 90 cases per 100,000 (1, 2). The majority of PAs, either secreting or non-functioning, are successfully treated by conventional surgery alone or in combination with medical treatment (3). Despite the benign form of PAs, a small number of patients with aggressive pituitary tumors (APTs) represent atypical morphological features of radiologically invasive growth, increased number of mitosis, extensive nuclear staining for p53, and Ki67>3% (4). In rare conditions, ~0.2% of pituitary adenomas are characterized as pituitary carcinomas (PCs), which elicit craniospinal and/or systemic metastases after initial diagnosis (5). Further courses of surgery and radiotherapy may partly palliate the symptoms, however, the complete disappearance of pituitary malignancies remains challenging (6). Currently, there are limited tumoricidal options for management of those life-threatening PAs.

Innovative strategies have been widely investigated to utmost reverse the malignant progression of PAs (7). Temozolomide (TMZ), an oral alkylating chemotherapeutic agent, has been established as first-line chemotherapy for high-grade gliomas and intracranial metastatic tumors (8). Encouraged by these findings, TMZ has been increasingly employed as salvage treatment for APT and PC patients after the failure of standard management with surgical, medical, and radiational treatments (9). TMZ has been demonstrated as a safe therapeutic agent offering a high clinical response rate in patients with APT and PC (10).

Nevertheless, several unaddressed issues exist in clinical employment of TMZ for APT and PC patients. Clinical outcomes vary between studies and have not been systematically estimated due to scarcity of data (11). Clinical benefit and optimal management for individuals with different baseline characters are still debating (12). Potential predictors affecting the clinical efficacy of TMZ have not been substantiated since the data were poorly documented (13, 14). A systematic review and meta-analysis exploring the definite clinical efficacy of TMZ in APT and PC patients are highly demanded. Herein, the present study aims to combine data from current large-scale retrospective studies of TMZ in patients with APT and PC, and thus gain more reliable estimations of specific outcomes and their relevant subgroups.

## Materials and Methods

### Selection Criteria

We selected the studies that met the following criteria: the manuscript was published in English, patients were diagnosed as APT or PC regardless of the baseline characteristics, TMZ was applied as first-line chemotherapy agent after conventional treatment, and specific outcomes of TMZ was mathematically or descriptively presented in the manuscript. Single case report or any studies not meeting those criteria were excluded.

### Search Strategy and Study Identification

For this meta-analysis, methods proposed in the Preferred Reporting Items for Systematic Review and Meta-analysis statement were in use. A literature retrieval was performed in PubMed, MEDLINE, Web of Science, and Cochrane Library for studies published up to December 31, 2020. Following MeSH terms were conditionally combined for online search: “temozolomide”; “pituitary adenoma,” “somatotroph adenoma,” “acromegaly,” “corticotroph adenoma,” “prolactinomas,” “lactotroph adenoma,” “gonadotroph adenoma,” “thyrotroph adenoma.” The study identification was extended to the reference list of included studies and relevant reviews. All of the aforementioned procedures were independently done by two reviewers (Mei Luo and Yiheng Tan). Any discrepancies were resolved by consensus within all co-authors.

### Data Extraction and Meta-Analysis

Data of interest include the demographic and clinical characteristics, prior treatment, histological features, radiological response, biochemical response, survival outcomes, and adverse events during TMZ employment in patients with APT and PC. Response assessment was universally defined as follow: complete response (CR) as the disappearance of all target lesions; partial response (PR) as a decrease of at least 30% of target lesions; stable disease (SD) as an insufficient shrinkage to qualify for PR, nor a sufficient increase to qualify for progression disease (PD); PD as a 20% increase or the appearance of one or more new lesions; the biochemical response was defined as >50% decrease of secreting hormone.

We collected data about MGMT expression and its promoter methylation status in surgical specimens from APT and PC patients, and correlated those molecular features with the radiological response to TMZ. MGMT expression was graded as minimal-expression group (≤ 10% MGMT immunoreactive cells), intermediate-expression (10–50% immunoreactive cells) and high-expression group (≥50% MGMT immunoreactive cells) according to immunohistochemistry in tissue sections. MGMT promoter methylation status was classified as methylated-group and unmethylated-group based on MSP. Additionally, data about the concomitant therapy of stereotactic and/or fractionated radiotherapy with TMZ were collected if available.

Data were synthesized by standard meta-analysis approach in StataSE 15 software. As a conservative and reliable systematic review, we utilized a random effect if the heterogeneity was obvious (I^2^ > 50% or *p* < 0.05), otherwise, a fixed effect was used. Statistically, *p* < 0.05 was considered as significant differences.

## Results

### Identification of Eligible Study

**Figure 1:**
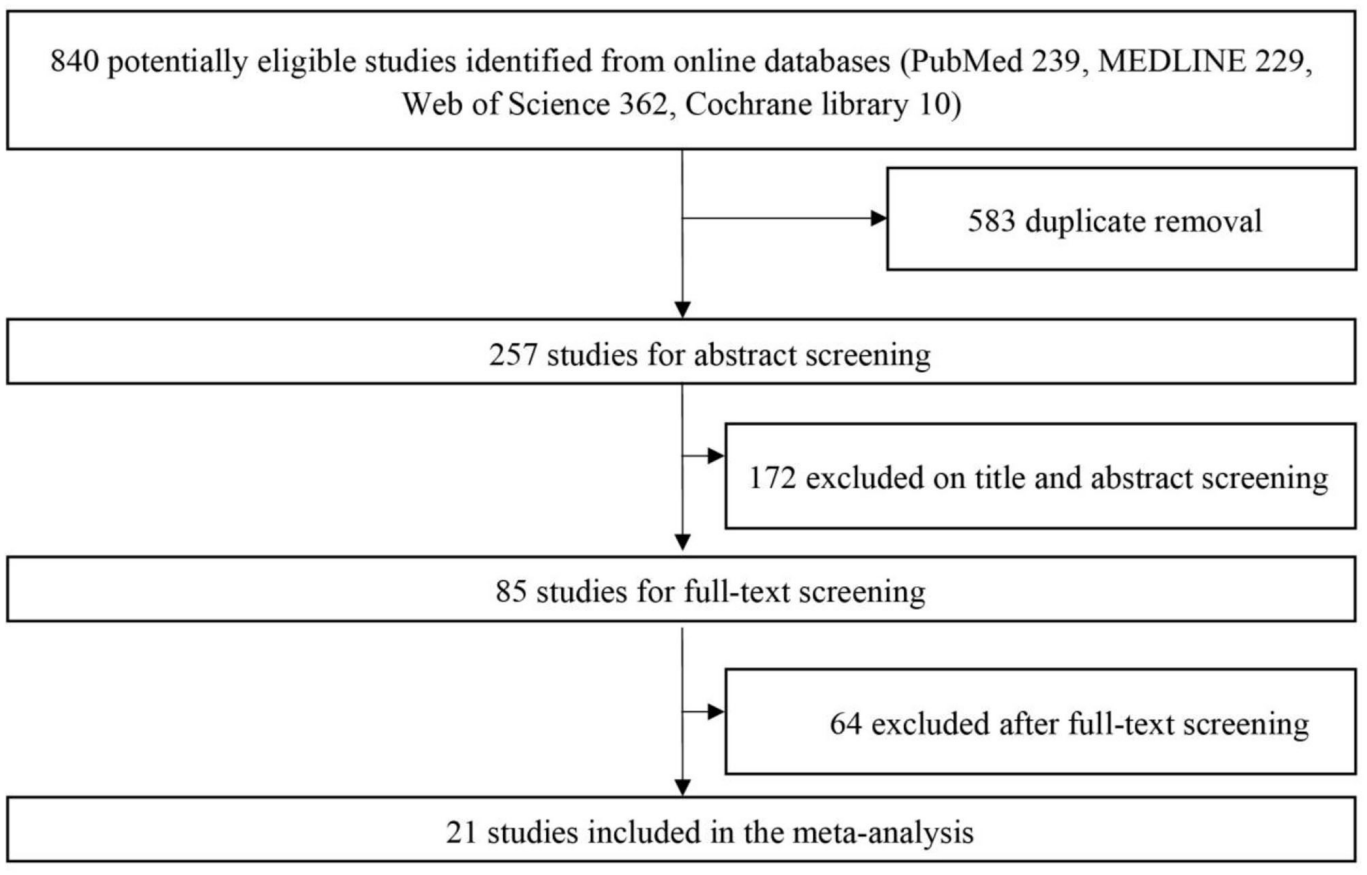
Algorithm of literature retrieval and study selection.

Initial identification of eligible studies generated 840 studies (239 from PubMed, 229 from MEDLINE, 362 from Web of Science, 10 from Cochrane Library). After duplicate removal of overlapping data, 257 studies were selected for abstract screening. With the detailed screening of title and abstract, 85 studies were included as relevant studies for full-text screening. Of the full-text articles retrieved, 21 studies met the preset inclusion criteria (10–12, 14–31). Reference list of included studies and relevant reviews did not provide any additional studies. An overview of the online search and selection algorithm was detailly illustrated in Figure 1.

The main features of all eligible studies were presented in Table 1. Twenty one studies from 12 countries were included. A total of 302 APT patients and 127 PC patients were involved in our meta-analysis. Most of the patients were treated with 150–200 mg/m^2^/day TMZ for 5 days every 4 weeks alone or in combination with capecitabine (CAPTEM), or 75 mg/m^2^/day TMZ given concurrently to radiotherapy.

**Table 1:** Main characteristics of eligible studies.

**References**	**Country/region**	**No. of patients**	**TMZ regimen**
Minniti et al. (15)	Italy	17 APTs and 4 PCs	75 mg/m^2^/day TMZ given concurrently to re-SRT, then 150–200 mg/m^2^/day for 5 days every 4 weeks or 50 mg/m^2^ daily for 12 months.
Lizzul et al. (16)	Italy	7 APTs and 1 PC	150–200 mg/m^2^/day for 5 days every 4 weeks.
Elbelt et al. (17)	Germany	34 APTs and 13 PCs	150–200 mg/m^2^/day for 5 days every 4 weeks for majority; 75 mg/m^2^/day for 3–6 weeks during radiotherapy followed by standard dosing in seven patients (“Stupp” protocol).
Santos-Pinheiro et al. (10)	The United States	17 PCs	150–200 mg/m^2^/day for 5 days every 4 weeks in six patients, or combined with capecitabine in two patients (CAPTEM), or concurrently with radiotherapy in one patient.
McCormack et al. (18)	European Society of Endocrinology	125 APTs, 40 PCs, and 1 unclassified	150–200 mg/m^2^/day for 5 days every 4 weeks for majority; 75 mg/m^2^/day for 6 weeks during radiotherapy followed by 6–12 months of standard dosing in six patients (“Stupp” protocol).
Jordan et al. (11)	The United States	4 APTs and 3 PCs	150–200 mg/m^2^/day for 5 days every 4 weeks.
Bengtsson et al. (14)	Sweden and Denmark	2 APTs and 3 PCs	150–200 mg/m^2^/day for 5 days every 4 weeks.
Lasolle et al. (19)	France	29 APTs and 14 PCs	150–200 mg/m^2^/day for 5 days every 4 weeks for majority; 75 mg/m^2^/day for 6 weeks during radiotherapy followed by standard dosing in six patients (“Stupp” protocol).
Losa et al. (20)	Italy	25 APTs and 6 PCs	150–200 mg/m^2^/day for 5 days every 4 weeks for majority; 75 mg/m^2^/day for 6 weeks during radiotherapy followed by standard dosing in two patients (“Stupp” protocol).
Aydogan et al. (12)	Turkey	3 APTs	150–200 mg/m^2^/day for 5 days every 4 weeks.
Ceccato et al. (21)	Italy	5 APTs	150–200 mg/m^2^/day for 5 days every 4 weeks.
Bruno et al. (22)	Argentina	6 APTs	140–320 mg/day for 5 days monthly for at least 3 months.
Bengtsson et al. (23)	Sweden, Denmark, Belgium, and Netherland	16 APTs and 8 PCs	150–200 mg/m^2^/day for 5 days every 4 weeks.
Zacharia et al. (24)	The United States	4 APTs	150–200 mg/m^2^/day for 5 days every 4 weeks in combination with capecitabine (CAPTEM)
Hirohata et al. (25)	Japan	3 APTs and 10 PCs	150–200 mg/m^2^/day for 5 days every 4 weeks.
Whitelaw et al. (26)	UK	3 APTs	150–200 mg/m^2^/day for 5 days every 4 weeks.
Raverot et al. (27)	France	3 APTs and 5 PCs	150–200 mg/m^2^/day for 5 days every 4 weeks.
Losa et al. (28)	Italy	5 APTs and 1 PC	150–200 mg/m^2^/day for 5 days every 4 weeks.
Bush et al. (29)	The United States	7 APTs	150–200 mg/m^2^/day for 5 days or 75 mg/m^2^/day for 21 days every 4 weeks.
Mohammed et al. (30)	Canada	3 APTs	150–200 mg/m^2^/day for 5 days every 4 weeks.
Fadul et al. (31)	The United States	2 PCs	150–200 mg/m^2^/day for 5 days every 4 weeks.

### Key Features of APT and PC Patients in TMZ Responders and Non-Responders

The following information of APT and PC patients were extracted from eligible studies if available: gender, age at diagnosis, age at TMZ starting, frequency of prior surgery and radiotherapy, APT and PC diagnosis, clinical subtype based on secretion type, histological features (Ki 67 index, p53 immunodetection, and presence of mitosis), and MGMT status (MGMT expression and its promoter methylation). The difference of interested parameters between responders and non-responders to TMZ were summarized in Table 2. The demographic features, age at enrollment, and prior treatment were not significantly varying between groups. The distribution of APTs and PCs was 77/35 in responders vs. 116/50 in non-responders (*p* = 0.841). Response rate was not different according to the histological features (Ki67 index, *p* = 0.151; p53 immunodetection, *p* = 0.075; mitosis, *p* = 0.146). Most of the parameters were not significantly linked with the radiological outcome of TMZ in patients of APT and PC, except for the clinical functioning subtype (*p* < 0.001) and MGMT expression level (*p* = 0.001), which deserved further analysis of their effect to alter the efficacy of TMZ.

**Table 2:** Key features of APT and PC patients in TMZ responders and non-responders.

**Characteristics**	**Non-responders**	**Responders**	***p*-value**
**Gender**, ***n***			0.439
Female	72	55	
Male	116	74	
Age at diagnosis^*^	42 [18–68]	47 [13–76]	0.562
Age at TMZ starting^*^	52 [22–78]	51 [18–70]	0.892
Prior surgery^*^	2 [0–4]	3 [0–5]	0.666
Prior radiotherapy^*^	1 [0–3]	1 [1–3]	0.884
**Diagnosis**, ***n***			0.841
Aggressive pituitary tumors	116	77	
Pituitary carcinomas	50	35	
**Clinical subtype**, ***n***			<0.001
Functioning	172	138	0.500
Corticotroph	88	68	
Gonadotroph	6	1	
Somatotroph	17	16	
Lactotroph	55	46	
Thyrotroph	6	7	
Non-functioning	57	10	
**Histological features**, ***n***			
Ki67 index			0.151
Ki67 <3%	27	10	
Ki67≥3%	106	69	
p53 immunodetection			0.075
p53 negative	66	45	
p53 positive	54	25	
Mitosis			0.146
Mitosis≥2/10 HPF	15	5	
Mitosis <2/10 HPF	3	3	
**MGMT status**, ***n***			
MGMT expression			0.001
Minimal expression	28	33	
Intermediate expression	6	5	
High expression	31	6	
MGMT promoter methylation			0.047
Promoter methylated	6	7	
Promoter unmethylated	20	7	

* *mean [range]*.

### Responsive, Survival, and Safety Outcomes of TMZ in APT and PC Patients

**Figure 2:**
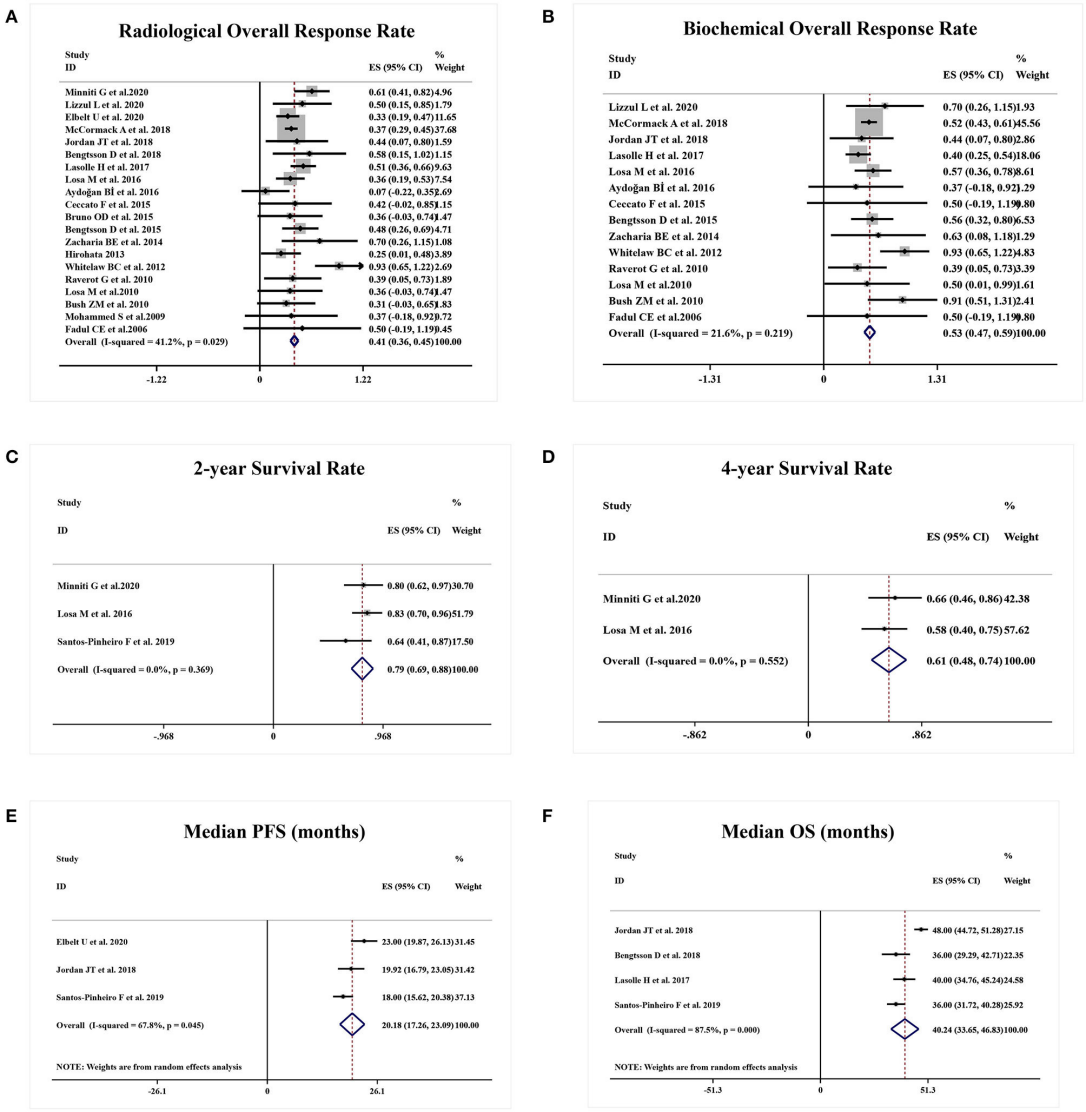
Responsive and survival outcomes of TMZ in APT and PC patients. **(A)** Radiological overall response rate was achieved in 41% of patients. **(B)** 53% of patients were biochemically responsive to TMZ. **(C,D)** 2-year survival rate was 79% and 4-year survival rate was 61%. **(E,F)** Median PFS and OS were 20.18 months and median OS was 40.24 months, respectively.

A total of 20 studies reported the radiological ORR. A sustained antitumor activities and radiological response were achieved in 41% APT and PC patients (95%CI 0.36–0.45, I^2^ = 41.2%, *p* = 0.029, Figure 2A). Likewise, 14 studies were eligible for biochemical ORR analysis, and 53% of patients (95%CI 0.47–0.59, I^2^ = 21.6%, *p* = 0.219, Figure 2B) were responsive to TMZ with a decrease of more than 50% hormone secretion.

A total of three studies presented the survival rate of APT and PC patients after TMZ employment. Two-year survival rate and 4-year survival rate were 79% (95%CI 0.69–0.88, I^2^ = 0.0%, *p* = 0.369, Figure 2C) and 61% (95%CI 0.48–0.74, I^2^ = 0.0%, *p* = 0.552, Figure 2D), respectively. Estimated median PFS from three studies was 20.18 months (95%CI 17.26–23.09, I^2^ = 67.8%, *p* = 0.045, Figure 2E) and median OS from four studies was 40.24 months (95%CI 33.65–46.83, I^2^ = 87.5%, *p* = 0.000, Figure 2F).

**Figure 3:**
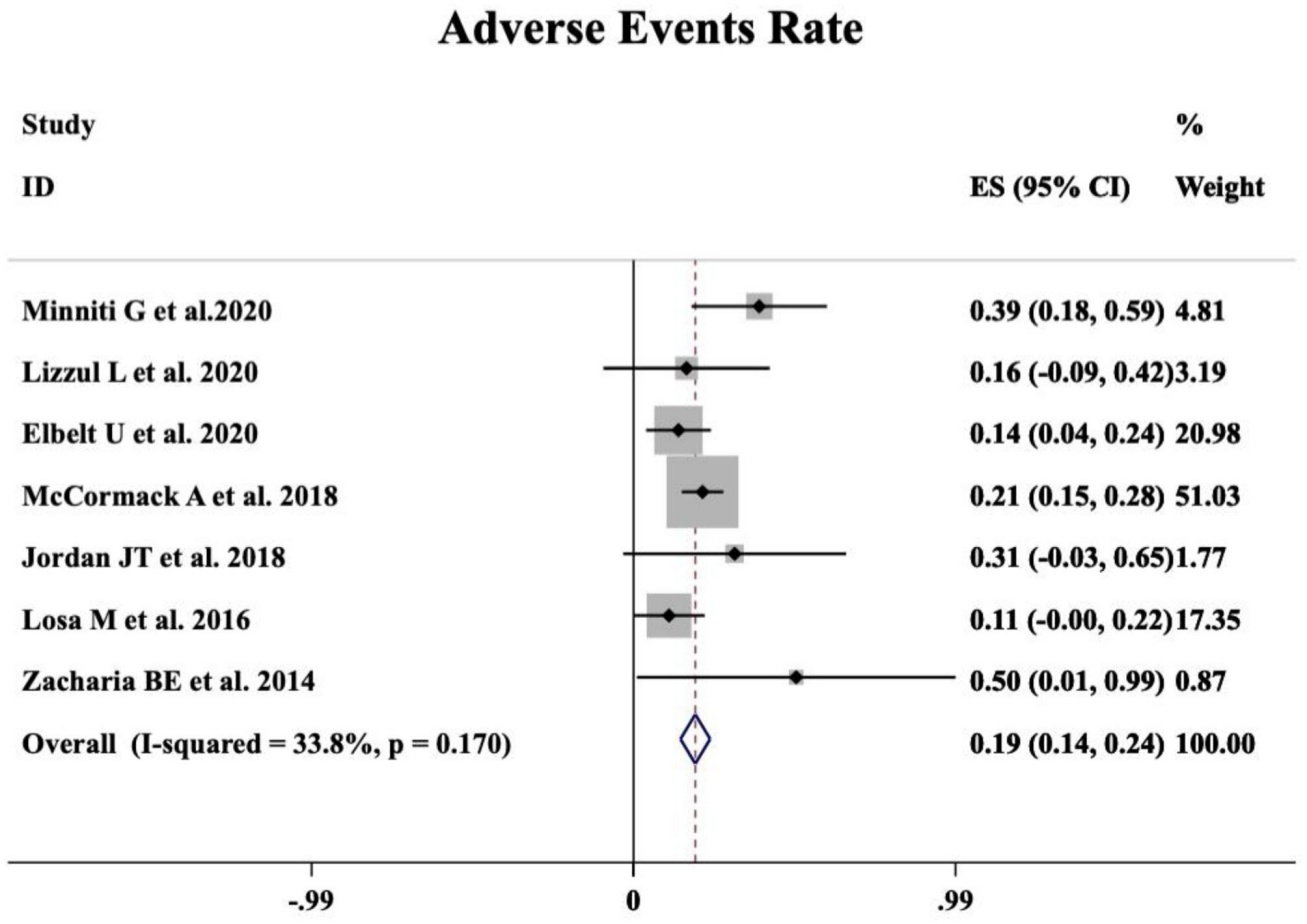
Occurrence of TMZ-related adverse events.

A meta-analysis was performed including seven studies that reported the safety profile of TMZ in APT and PC patients. Grade 2–4 TMZ-related adverse events moderately occurred in 19% patients (95%CI 0.14–0.24, I^2^ = 33.8%, *p* = 0.170, Figure 3).

### Predictive Role of MGMT Status for TMZ Efficacy

**Figure 4:**
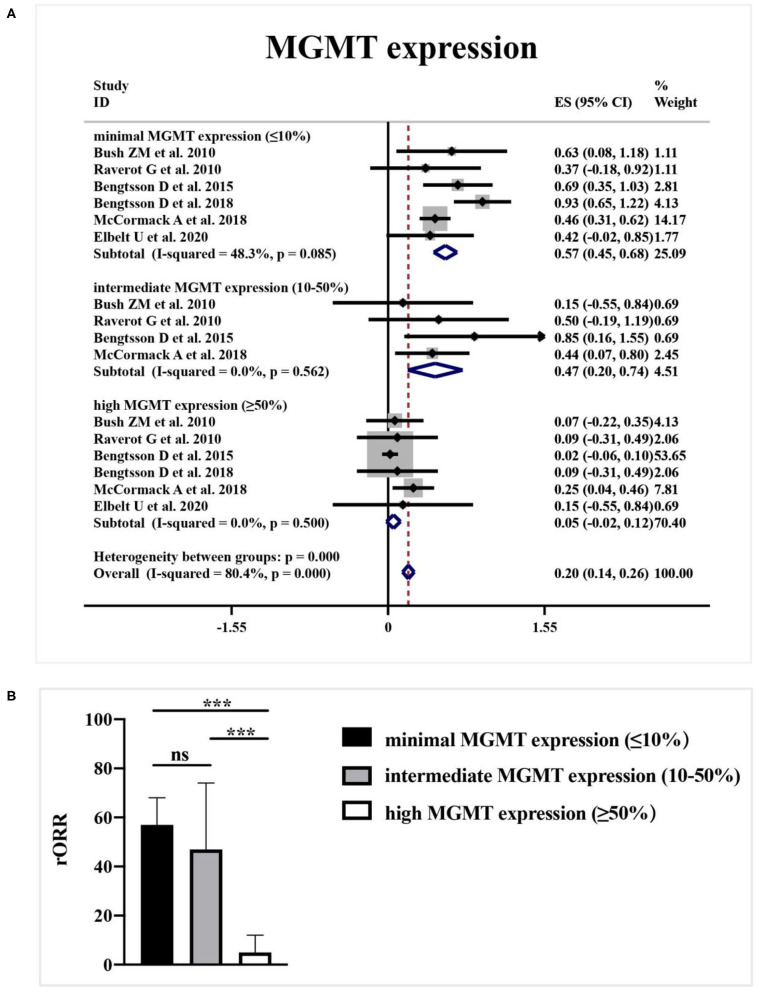
The correlation between MGMT expression level and TMZ radiological response. **(A)** Radiological response rate was 57% in patients with minimal-MGMT expression, 47% in patients with intermediate-MGMT expression, and 5% in patients with high-MGMT expression. **(B)** Radiological response rate was spectacularly lower in APT and PC patients with high-MGMT expression than those with minimal and intermediate-MGMT expression group in the quantitative histogram. ^***^P < 0.001.

**Figure 5:**
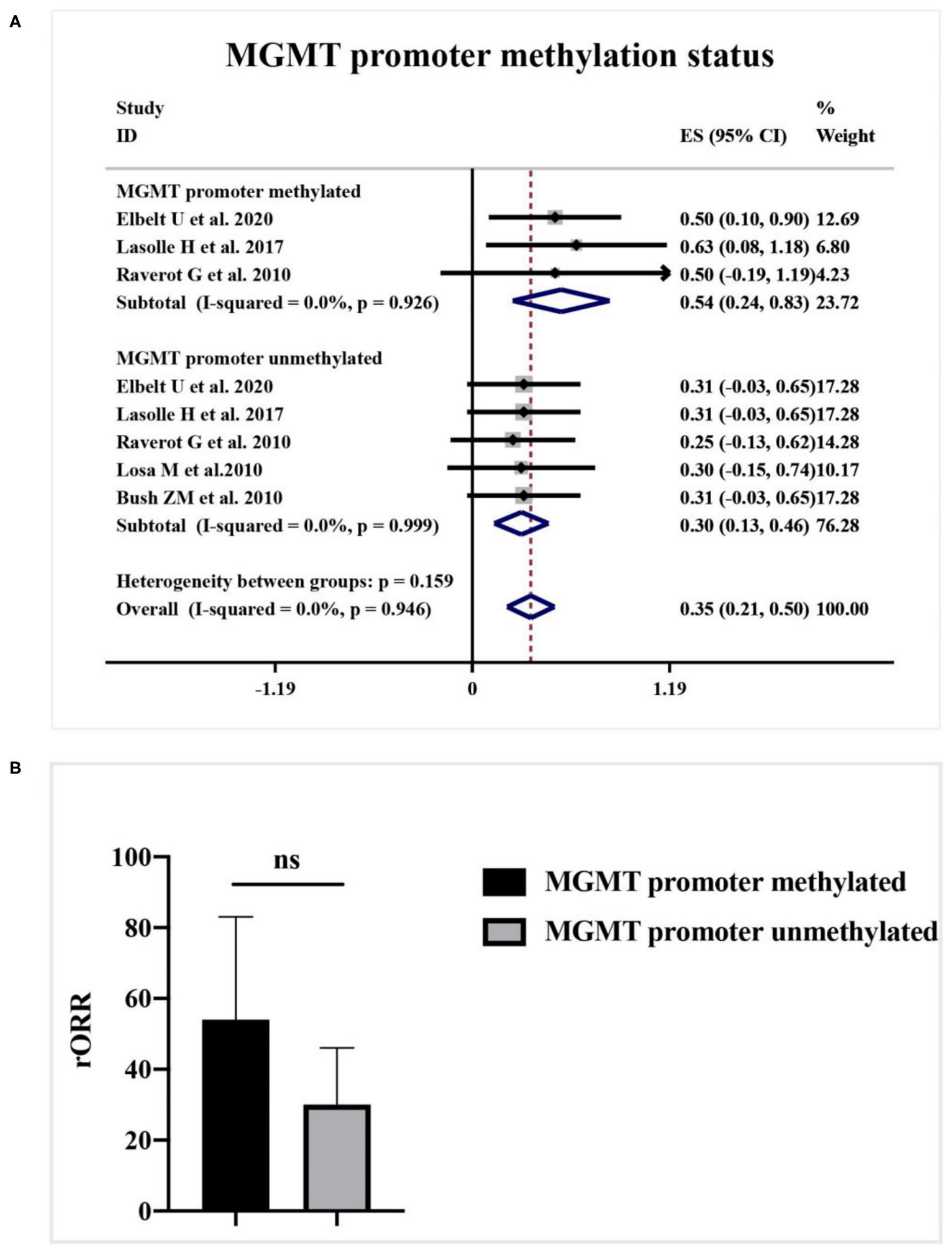
The correlation between MGMT promoter methylation status and TMZ radiological response. **(A)** Radiological response rate was 54% in the MGMT promoter methylated group, and 30% in the unmethylated group. **(B)** The quantitative histogram showed the difference between groups was not significant.

As shown in Figure 4A, a meta-analysis revealed that radiological response rate was spectacularly lower in APT and PC patients with high-MGMT expression (0.05, 95%CI 0.00–0.12) than those with minimal-MGMT expression group (0.57, 95%CI 0.45–0.68) (*p* < 0.001) and intermediate-MGMT expression group (0.47, 95%CI 0.20–0.74) (*p* = 0.004), while the difference between minimal- and intermediate-MGMT expression group was not significant in Figure 4B (*p* = 0.503). Relatively, even though the rORR of TMZ in involved patients was higher in the MGMT promoter methylated group (0.54, 95%CI 0.24–0.83) than unmethylated (0.30, 95%CI 0.13–0.46) in Figure 5A, the difference between groups was not as striking as MGMT expression analysis (*p* = 0.159, Figure 5B).

### Correlation Between the Clinical Subtype of Hormone Secretion and TMZ Efficacy

**Figure 6:**
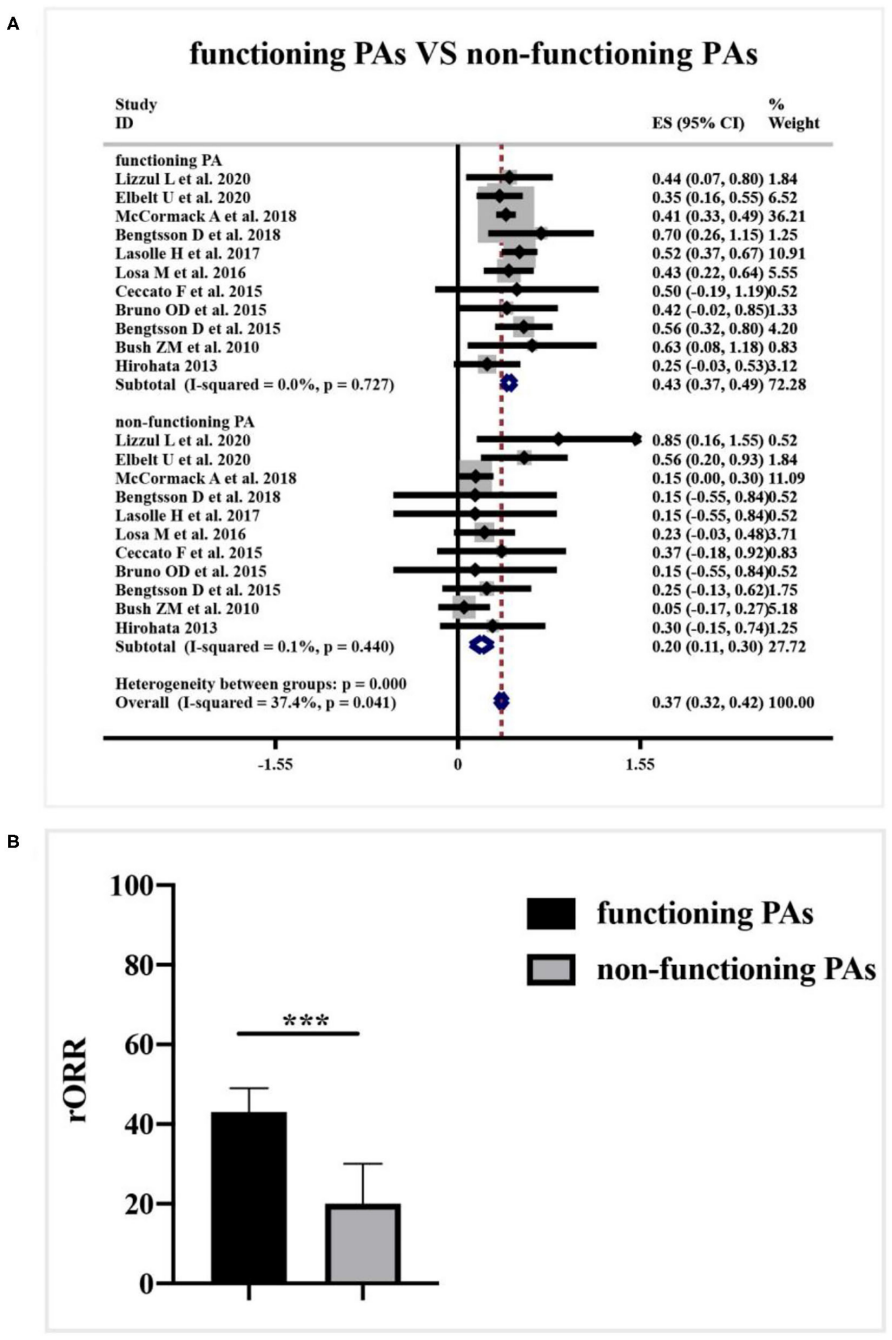
Relationship between clinical functioning subtype and radiological response of TMZ in patients with APT and PC. **(A)** 43% radiological response rate to TMZ presented in the functioning subset, and non-functioning specimens only 20% radiological response. **(B)** The difference between groups was not dramatic as shown in the quantitative histogram. ^***^P < 0.001.

A subgroup analysis of radiological response was performed based on the functioning subtype of APT and PC patients in Figure 6A. Comparing with 43% (95%CI 0.37–0.49, I^2^ = 0.0%, *p* = 0.727) clinical response rate to TMZ in functioning subset, non-functioning specimens only generated 20% (95%CI 0.11–0.30, I^2^ = 0.1%, *p* = 0.440) radiological response rate. TMZ tended to be more effective in patients with functioning APT and PC (*p* < 0.001, Figure 6B).

### Concomitant Treatment of Radiotherapy and TMZ

**Figure 7:**
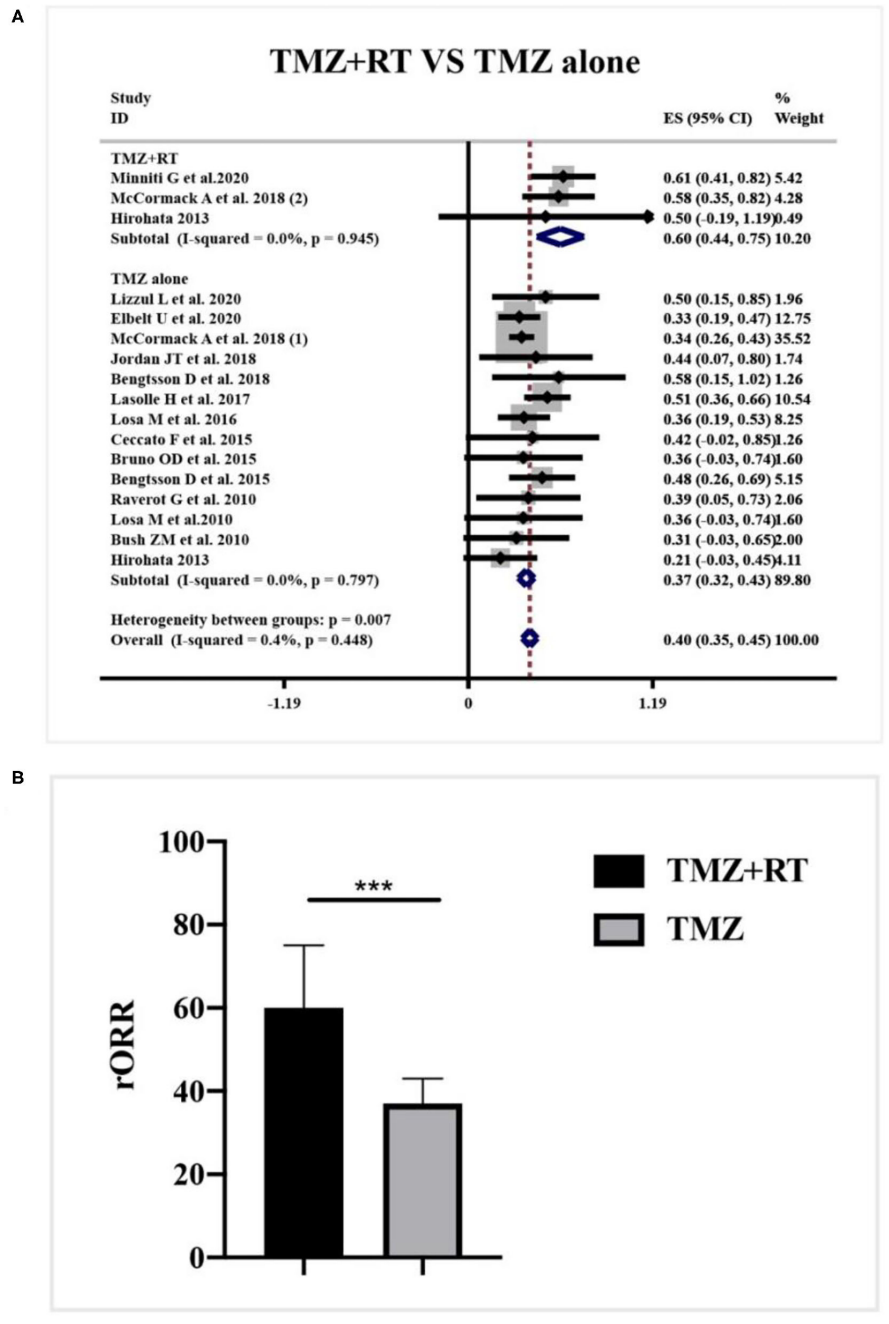
Radiological response after concomitant application of radiotherapy and TMZ for patients with APT and PC. **(A)** Concomitant application of radiotherapy and TMZ generated 60% radiological response, and TMZ monotherapy elicited a 37% radiological response. **(B)** Quantitative histogram showed the combined therapy significantly increased the radiological response than TMZ monotherapy. ^***^P < 0.001.

Data about the concomitant application of radiotherapy and TMZ was reported in 3 studies. According to a meta-analysis in Figure 7A, the clinical employment of TMZ monotherapy elicited a 37% rORR (95%CI 0.32–0.43, I^2^ = 0.0%, *p* = 0.797), while combined radiotherapy and TMZ dramatically increased the rORR to be 60% (95%CI 0.44–0.75, I^2^ = 0.0%, *p* = 945) (*p* = 0.007, Figure 7B).

## Discussion

The present meta-analysis substantially confirms the antitumoral activities of TMZ for patients with APT and PC. We illustrate that the low MGMT expression and hormone-secreting function may work as predictors for better TMZ response. Furthermore, the concomitant application of radiotherapy and TMZ can ameliorate the TMZ response and tend to be a promising salvage treatment in those not responding to initial TMZ monotherapy.

According to the WHO classification system for pituitary tumors, benign form of pituitary adenomas and pituitary carcinomas are easily categorized. However, APT is often defined according to its clinically aggressive behaviors, with earlier and more frequent recurrence or progression under conventional therapy (1, 4). It cannot be either located as benign or malignant adenomas, but should be considered as an intermediate form. The vaguely defined criteria of APT require more reliable histopathological features to predict its clinical behaviors. However, the correlation between current atypical histopathology (ki67 > 3%, p53 expression, and increased mitotic numbers) and its clinical behaviors remain debatable (32, 33). Emerging biomarkers to facilitate the early predictions of clinically aggressive behaviors and effectiveness of treatment are still requiring. In our study, diversified biomarkers have been investigated to confirm its predictive role in the effectiveness of TMZ employment, which may also be utilizable for the prediction of aggressive behaviors in APT.

As the first-line chemotherapy regimen, TMZ has documented its safety and efficacy for progressive pituitary adenomas (29). However, the lack of guidelines for clinical management induces significant heterogeneity in the use of TMZ for those rare pituitary tumors (22). Diversified factors are involved in the resistance of TMZ chemotherapy in individuals (14). For instance, the demographic features before chemotherapy and histological characters of proliferation may alter the efficacy of TMZ. The alkylating action of TMZ are resulted from epigenetic modification of DNA by methylation of gene promoter sites, thus disrupt the protein expression of the cell cycle (21). Hence, proliferative markers might be potential predictors for TMZ response (34). In our study, the demographic parameters, prior surgery and radiotherapy, histological features, biological factors, and other interested biochemical maters in responders and non-responders to TMZ have been systematically reviewed. As our results showed, none of them are determined as predictors of TMZ response.

Preliminary data proposed that DNA repair enzyme O6-methylguanine DNA methyltransferase (MGMT) expression was correlated with the clinical benefit of TMZ in APT and PC patients (35). According to our previous research, the majority of prolactinomas showed minimal MGMT expression, which provide a rational for the utility of TMZ to manage the aggressive prolactinomas (36). MGMT may attenuate the effect of TMZ by removing additional alkyl groups. The lack of MGMT expression would be linked with damaged DNA repair capacity and thus predicts the clinical efficacy of TMZ (26). Nevertheless, some studies introduce that MGMT may not be significantly associated with efficacy of TMZ chemotherapy in APTs and PCs (37). According to the synthesized data, our meta-analysis concludes that low MGMT staining predict a favorable response to TMZ therapy. Besides, relied on clinical experience and in line with our published findings, it is important to be noted that little is known about the variation of MGMT expression during the tumor progression (38). In rare, MGMT expression patterns will change during the first and last surgery. And even within the identic biopsy, patients exhibit a heterogeneous pattern of MGMT expression among tumor cells (23). Therefore, the present pattern of MGMT expression in the biopsy is increasingly recommended for patient selection during TMZ employment.

MGMT promoter methylation status, which can epigenetically alter the gene expression of MGMT, may also be correlated with TMZ response (39). Currently, the exact influence of MGMT promoter methylation status on chemotherapy in APT and PC patients is still questioned (28). In our study, patients with methylated MGMT promoter generate a higher radiological response, but the difference is not as significant as the effect of MGMT expression. It implies that MGMT expression level is not only affiliated with the promoter methylation status but also regulated by other unique epigenetic and transcriptional microenvironment factors (28, 29, 36). Moreover, the MGMT promoter methylation is simply classified as the positive group and the negative group without a cutoff of grading as definite as the gene expression, which might be the principal event for the reduction of correlation between MGMT promoter methylation and TMZ response (20). These hypotheses may partially explain the discordance between MGMT promoter methylation and MGMT expression (29, 40). Herein, it seems too early to conclude on any correlation between MGMT expression and promoter methylation, or between the presence of methylation and response to temozolomide.

Another new finding in this study is that clinical subtypes of secreting function may also work as a predictive factor for TMZ response in patients with APT and PC. The predominance of functioning PA in TMZ responders may reflect its tendency of proliferation and invasiveness (19). Nonetheless, little is known about the mechanisms and detailed biological process in this finding. Over-secretion of the involved hormone may not only reflect the clinical manifestations but also be involved in the pathogenesis and TMZ resistance for APT and PC patients, which needs further investigation in future studies (18).

From current knowledge, TMZ application can exhibit a 40% radiological response rate with rare and mild adverse events, which can be easily controlled by pre- or post-medications (17). It was kindly suggested to extend the duration of TMZ application, in case of the progression in advance (16). The majority of APT and PC patients received continuous TMZ therapy. Whereas, the discontinuation of TMZ exists due to the severe adverse events, early deterioration, and insufficient therapy adherence (10). The second course of TMZ is often feeble to generate clinical efficacy and other options after discontinuation is scarce (16, 41). As for those not responsive to TMZ alone, recent evidence suggests the potential benefit of concomitant therapy of radiotherapy with TMZ (18). Our study confirms that concomitant chemoradiotherapy can improve the radiological response rate from 37 to 60%. Combined treatment subsequently increases the toxicity, noteworthy, the TMZ-related adverse events for single medicine remain unchanged, which will not induce the dose delaying and discontinuation of TMZ (25). Therefore, the potential benefit of combined chemoradiotherapy is warranted in future prospective trials (15).

Clinically functioning PA, low MGMT expression, and concomitant radiotherapy are associated with a better radiological response of TMZ. However, the limited long-term effect of TMZ and poor efficacy of other drugs demonstrate the necessity of more innovative strategies for treatment of APT and PC (42). The clinical efficacy of additional cytotoxic chemotherapy agents, such as carboplatin, cisplatin plus etoposide, cyclophosphamide et al., are still unclear. The rest of patients with unmet management of tumor progress still requires more effective treatment than TMZ (43). Nowadays, diversified innovative agents, including immune checkpoint inhibitors, VEGFR-targeted therapy, PI3K/AKT/mTOR inhibitors, and tyrosine kinase inhibitors, represent inspiring clinical benefit among those patients under TMZ alone or in combination therapy (7, 44, 45).

Limitations of this meta-analysis should be concerned. The results and conclusion should be conservative owing to the retrospective nature of included studies (30). Besides, the survival outcomes of TMZ in APT and PC patients are objective without placebo control, whether radiological and biochemical outcomes of TMZ can translate to be better survival outcomes are still controversial (11). In the future, large-scale prospective clinical studies, possibly through a multicenter collaboration, are required to further determine our findings.

## Conclusion

In conclusion, our meta-analysis illustrates the accurate response effect of TMZ in APT and PC patients resistant to conventional treatments. These findings underline the adherence of guideline on the clinical employment of TMZ and management of pituitary malignancies. MGMT expression status and clinical subtype of secreting function should be defined before the start of TMZ, so as to predict the prognosis in advance for PAs. In particular, combined therapy of radiotherapy with TMZ will be beneficial for patients not responsive to TMZ monotherapy.

## Data Availability Statement

The raw data supporting the conclusions of this article will be made available by the authors, without undue reservation.

## Author Contributions

ML, YT, DZ, and HJ: project design. ML, YT, WC, BH, CD, and ZW: data collection and analysis. ML, YZ, and HW: manuscript preparation and revision. All authors contributed to the article and approved the submitted version.

## Conflict of Interest

The authors declare that the research was conducted in the absence of any commercial or financial relationships that could be construed as a potential conflict of interest.
